# Priority areas for cannabis and cannabinoid product research in South Africa

**DOI:** 10.4102/phcfm.v10i1.1711

**Published:** 2018-06-21

**Authors:** Tanya N. Augustine, Carel J. Cairns, Sean Chetty, Lisa G. Dannatt, Nadine Gravett, Glenda Grey, Gerhard Grobler, Zukiswa Jafta, Peter Kamerman, John Lopes, Motlalepula G. Matsabisa, Pierre Mugabo, Michelle Mulder, Charles Parry, Solomon Rataemane, Nandi Siegfried, Vanessa Steenkamp, Eileen Thomas, Richard van Zyl-Smit

**Affiliations:** 1 School of Anatomical Sciences, Faculty of Health Sciences, University of the Witwatersrand, South Africa; 2 Department of Anaesthesia, Grey’s Hospital, Pietermaritzburg, South Africa; 3 Department of Anaesthesiology and Critical Care, Faculty of Medicine and Health Sciences, Stellenbosch University, South Africa; 4 Department of Psychiatry and Mental Health, Faculty of Health Sciences, University of Cape Town, South Africa; 5 South African Medical Research Council, Cape Town, South Africa; 6 Department Psychiatry, Faculty of Health Sciences, University of Pretoria, South Africa; 7 Frere Hospital, East London, South Africa; 8 Brain Function Research Group, Faculty of Health Sciences, University of the Witwatersrand, South Africa; 9 School of Biomedical Sciences, Curtin University, Australia; 10 Department of Biomedical Sciences, Faculty of Medicine and Health Sciences, Stellenbosch University, South Africa; 11 Department of Pharmacology, Faculty of Health Sciences, University of Free State, South Africa; 12 School of Pharmacy, Faculty of Natural Sciences, University of the Western Cape, South Africa; 13 Grants Innovation and Product Development, Strategic Health Innovation Partnerships, South African Medical Research Council, South Africa; 14 Alcohol and Tobacco and Other Drug Research Unit, South African Medical Research Council, South Africa; 15 Department of Psychiatry, Faculty of Medicine and Health Sciences, Stellenbosch University, South Africa; 16 Department of Psychiatry, School of Medicine, Sefako Makgatho Health Sciences University, South Africa; 17 Department of Psychiatry and Mental Health, Faculty of Health Sciences, University of Cape Town, South Africa; 18 Department of Pharmacology, Faculty of Health Sciences, University of Pretoria, South Africa; 19 Department of Psychiatry, Faculty of Medicine and Health Sciences, Stellenbosch University, South Africa; 20 Lung Institute and Division of Pulmonology, Faculty of Health Sciences, University of Cape Town, South Africa

The legalisation of cannabis for medicinal use is a contentious space both politically and in the medical community. In 2014, the Medical Innovation Bill introduced by Mario Oriani-Ambrosini MP, aimed to shift the political and legal positions of cannabis as an illegal substance to one available for research and medical use. To date, progress on this has been slow. Cannabis and cannabinoid products are currently available for medicinal use in several countries, including the Netherlands and 29 states in the United States. Locally, anecdotal reports suggest that many of our patients with chronic medical conditions are using cannabis and cannabis-derived or cannabinoid products for symptom alleviation.

In January 2016, the Alcohol, Tobacco and Other Drug Research Unit (ATODRU) of the South African Medical Research Council (SAMRC) produced a policy brief evaluating the findings of a published systematic review of current evidence to inform the discussion.^1,2^ The review identified moderate quality evidence to support the use of medicinal cannabis for chronic pain, chemotherapy-induced nausea, vomiting, and multiple sclerosis,^1,2^ but the many formulations, dosages and routes of administration limit recommendations.

Subsequently, the SAMRC brought key stakeholders representing all nine faculties of health sciences at South African universities together on 08 February 2017 at the SAMRC Medicina Campus, Cape Town, to explore opportunities for conducting local research and clinical trials of medicinal cannabis and cannabinoids to inform local policy-making. The workshop identified three major research priorities:

- Conduct a national, multisite clinical trial of cannabinoids following identification of the optimal formulations, dosage and relevant clinical indications from the current evidence base to inform trial protocol development.
- Support exploratory research to quantify the prevalence and qualify the current use of extracts (e.g. oils) in the community to alleviate pain and other symptoms; methods to include are as follows, (1) community- or clinic-based cross-sectional surveys or online survey of general public, (2) cross-sectional surveys of practising general practitioners and/or specialists in pain clinics with respect to their knowledge of patient use of cannabis extracts through patient disclosure.
- Conduct qualitative evaluation(s) of possible barriers and facilitators to medical practitioners prescribing cannabis or cannabinoids for medicinal purposes, should it be legalised in the future.

Further to this meeting, an SAMRC-supported team of researchers evaluated the evidence for a trial of a cannabinoid for the management of pain related to HIV associated sensory neuropathy (HIV-SN). The team selected painful HIV-SN as the condition based on: (1) the high burden of HIV and HIV-SN in the South African population, despite availability of improved antiretroviral drug regimens, and (2) the absence of empirical evidence supporting the efficacy of pharmacological agents typically recommended for the management of neuropathic pain in this population group. They limited their assessment to high-quality trials of greater than 12 weeks duration, included participants with at least moderate intensity pain at baseline and who met current recommendations for trial design in the field of chronic pain.^3^ Only three studies, all using a fixed dose combination of tetrahydrocannabinol (THC) and cannabidiol (CBD) delivered via oromucosal spray were identified, and the results of the GRADE^4^ assessment are shown in Table 1.

**TABLE 1:** GRADE assessment for nabiximol versus placebo for chronic pain.

Quality assessment							Number of patients				Effect			Quality	Importance
Number of studies	Design	Risk of bias	Inconsistency	Indirectness	Imprecision	Other considerations	Cannabis		Placebo		Relative %	95% CI	Absolute		
							*n*	%	*n*	%					
3†	Randomised trials	Serious‡	Number of serious inconsistencies§	No serious indirectness	Number of serious imprecisions	None	172/439	39.20	155/437	35.50	RR 1.13	0.86% –1.5%	46 more per 1000 (from 50 fewer to 177 more)	Moderate	Critical

Note: Pain relief (follow-up: 14 weeks –15 weeks).

† , Included studies: GW Pharma (EudraCT number: 2004-002530-20): 297 patients with diabetic peripheral neuropathy; Langford et al.6: 339 patients with multiple sclerosis; Serpell et al.7: 303 patients with at least one of the following underlying conditions which caused their peripheral neuropathy: post-herpetic neuralgia, peripheral neuropathy, focal nerve lesion, radiculopathy or complex regional pain syndrome (CRPS) type 2. All trials evaluated the effects of Sativex (THC or CHB oromucosal spray);

‡ , Risk of bias: Graded as serious. High risk of attrition in GW Pharma. Allocation concealment unclear in Langford and Serpell;

§ , Inconsistency: I^2^ was 47.6% indicating moderate heterogeneity. However, we did not downgrade the inconsistency as the inconsistency may be explained by the different population groups included in each trial.

The team concluded that clinical equipoise^5^ exists for a fixed dose THC:CBD oromucosal cannabinoid spray trial for the management of painful HIV-SN. This was based on the GRADE assessment, the absence of demonstrably effective alternative therapies for painful HIV-SN and the clinical need in people living with HIV. The team is currently working on a study protocol for a multisite trial that conforms with the current highest standards for trials of analgesic agents for chronic pain.^3,5^ The SAMRC will be releasing a Request for Applications (RFA) from groups interested in participating in trials in due course.

In medical practice, there is a balance between early adoption of new innovation and a slower, more considered approach prioritising evidence and safety over potential benefit. Though there are some clear areas where evidence supports the use of medicinal cannabis (as reviewed in the SAMRC policy brief), this should not imply that cannabis is a panacea for all ails or that it should be freely available without due control of production quality, disease indications and specific dosing recommendations. Research is urgently needed and regulatory changes will promote and allow for high priority, locally relevant research using regionally sourced products.

It is imperative that the medical and scientific community – both practitioners and researchers – are involved in informing and developing evolving cannabis policies. Much confusion exists between medical versus recreational usage, the legal statutes of medical and recreational possession and usage, and the scientific evidence compared to impassioned anecdotal reports of its efficacy in addressing a host of medical conditions. We owe it to our patients to offer the best relief of suffering with all available therapies based on the rational assessment of benefit, cost and potential harms of cannabis and cannabinoid products for medicinal use.
